# Hematology, Clinical Biochemistry, and Blood Cell Morphology Features of Captive *Bothrops jararaca* From Southeast Brazil

**DOI:** 10.1111/vcp.70032

**Published:** 2025-08-25

**Authors:** Amanda O. Alcantara, Bianca M. M. Reis, Juliana G. Fonseca, Maíra A. C. Sousa, Vitor Hugo A. Roxo, Guilherme J. Souza, Fúlvia de Fátima A. Castro, Guilherme N. Souza, Jorlan Fernandes, Elba Regina S. Lemos, Aline M. Souza

**Affiliations:** ^1^ Faculdade de Veterinária Universidade Federal Fluminense Niterói Brazil; ^2^ Instituto Vital Brazil Niterói Brazil; ^3^ Departamento de Medicina Veterinária Universidade Federal de Juiz de Fora Juiz de Fora Brazil; ^4^ Empresa Brasileira de Pesquisa Agropecuária Juiz de Fora Brazil; ^5^ Laboratório de Hantaviroses e Rickettsioses Instituto Oswaldo Cruz/FIOCRUZ Rio de Janeiro Brazil

**Keywords:** blood cell count, clinical chemistry, hemoparasite, *Hepatozoon*, snake, Viperidae

## Abstract

**Background:**

Snakebite envenomation is a worldwide public health issue, particularly relevant to low and middle‐income countries. *Bothrops* spp. antivenom is needed for snakebite treatment, which requires the maintenance of healthy snakes in captivity. Hematology and clinical biochemistry are important tools for monitoring the health status of these animals, as well as detecting hemoparasites (i.e., *Hepatozoon* spp.).

**Objectives:**

This study aims to establish hematologic and biochemical reference intervals in a population of captive *B. jararaca* in a Brazilian serpentarium and assess blood cell morphology.

**Methods:**

Blood samples of 32 specimens of captive *B. jararaca* were collected and analyzed for complete blood count and biochemical variables, such as total proteins, albumin, cholesterol, triglycerides, calcium, phosphorus, urea, creatinine, uric acid, aspartate aminotransferase, alanine aminotransferase, alkaline phosphatase, gamma‐glutamyl transferase, and creatine kinase. Blood smears were also evaluated to investigate blood parasites. Each variable was statistically analyzed according to the ASVCP guidelines and compared between sexes and the occurrence of *Hepatozoon* spp.

**Results:**

This study establishes the hematology and clinical biochemistry reference intervals and describes *B. jararaca* blood cell morphology. We observed no differences between sexes.

**Conclusions:**

Our study provides the first reference intervals for hematologic and biochemical variables for *B. jararaca* on the basis of ASCVP recommendations. The RI described could be essential for the management and treatment of *B. jararaca* kept under similar captive conditions.

## 1 Introduction

Snakebite envenomation is considered a neglected tropical disease according to the World Health Organization [1]. In Brazil, where the genus *Bothrops* is responsible for the majority of snakebite in the country, in particular, *Bothrops jararaca* (Wied‐Neuwied, 1824), endemic to South America in Brazil's Southern and Southeastern region, associated with the Atlantic forest, and in some regions of Paraguay and Argentina bordering Brazil [2]. The snakebite envenoming treatment depends on the production of antivenom in institutions that keep different species of snakes in captivity [3]. In this context, hematology is an important diagnostic tool for the clinical evaluation of captive snakes [4]. Despite the description of variables in some species of the genus *Bothrops* [5, 6, 7, 8], there is only one report of hematologic values for *B. jararaca* with a smaller sample size (*n* = 10) [7] and no studies are available on biochemical analytes.

Intrinsic and extrinsic factors can affect hematologic and biochemical variables, including parasitism [9]. Hemogregarines are a diverse group of intracellular parasites that can be found in reptiles. Members of the genus *Hepatozoon* (Miller, 1908) are the most identified intracellular protozoa parasitizing erythrocytes from free‐living and captive snakes [10, 11, 12]. Infections with *Hepatozoon* spp. are typically acquired through horizontal transmission by ingestion of infected invertebrates or intermediate prey. The occurrence of captive snakes infected with *Hepatozoon* spp. may be associated with the parasite's longevity within its host rather than the potential for transmission or reinfection in captivity [12], as these agents are well‐adapted to their hosts and cause minimal pathological changes [13]. Although some studies do not indicate hematological abnormalities associated with *Hepatozoon* spp. infection [14], accelerated destruction of erythrocytes was reported in infected snakes [15].

The study aimed to propose reference intervals (RIs) for hematologic and serum biochemical variables from *B. jararaca* snakes kept in captivity within a research institution and assess blood cell morphology.

## 2 Material and Methods

### 2.1 Sample Collection

This study was authorized by the Chico Mendes Institute for Biodiversity Conservation (ICMBio), through the SISBIO system (protocol A42BC58), and approved by the Animal Ethics Committee of the Vital Brazil Institute (CEUA‐IVB, protocol 021/21).

The snakes were screened from January to August 2023 and were held captive in the serpentarium of Vital Brazil Institute (22°54′21″ S 43°05′49″ W), located in the city of Niterói in the state of Rio de Janeiro, Brazil. The animals were rescued by the environmental police from different municipalities in the state of Rio de Janeiro and were maintained in captivity for venom extraction, with adequate sanitary management and constant monitoring by veterinarians and biologists. The snakes had been held in captivity for between 2 months and 7 years, individually or in pairs, in rectangular boxes of polypropylene in a room with free access to water and at room temperature (24°C–28°C). All the snakes were previously sexed by probing. The temperature and humidity of indoor environments were monitored. Once a month, they were regularly fed with rodents.

The physical examination was conducted [16], and the animals were required to meet the following criteria: have an ideal body condition score, show normochromic oral mucosa, be well‐hydrated, not have an infestation of ectoparasites, undergo complete ecdysis, and exhibit normal behavior, being responsive and alert to the environment and handling. Snakes with hemoparasites detected in their blood smears were excluded from the determination of the RI. All animals were adult, and pregnant females were not included. All snakes were fasted for two to 3 weeks before blood collection.

A total of 32 *B. jararaca* snakes (25 females and 7 males) were included in the study, with all 32 sampled for hematological analysis and 30 for biochemical evaluation (23 females and 7 males). All the snakes were sampled in the morning. The snakes were physically restrained with restraint tubes, and the blood was collected from the cervical paravertebral sinus or ventral coccygeal vein [17] with a 3 mL syringe and 24G ¾ (20.0 × 5.5 mm) needle. A total volume of 1 to 2 mL of blood from each snake was drawn and stored in a K2‐EDTA microtube (0.5 mL) for complete blood count (CBC) and serum separating gel and clot activator tube (1.0–1.5 mL) for biochemistry tests. Blood collection was performed by an experienced veterinarian. Pre‐analytical care included rapid refrigeration, which was initiated after clot retraction to prevent hemolysis. Samples were transported under refrigeration within 3 h to the Veterinary Clinical Pathology Laboratory (LABHUVET) of the Federal Fluminense University.

### 2.2 Hematological Analysis

Erythrocyte, leukocyte, and thrombocyte counts were determined manually using a hemocytometer (Neubauer chamber) with a Natt and Herrick stain (diluted 1:100) [4]. The packed cell volume (PCV) was obtained with the microhematocrit method after centrifugation for 5 min at 14 490 g (Microhematocrit centrifuge, SPIN 1000, Microspin, Brazil). Hemoglobin concentration (Hb) was measured using a colorimetric method (Hemoglobin, Labtest Diagnóstica S. A., Minas Gerais, Brazil) by the cyanmethemoglobin method in a semi‐automated analyzer (Bio 2000, Bioplus, São Paulo, Brazil). Hematimetric indices, such as the mean corpuscular volume (MCV) and mean corpuscular hemoglobin concentration (MCHC) were calculated using standard formulas. The total plasma protein (TTP) concentration was estimated by refractometry (Manual refractometer RHC‐200, Megabrix, Paraná, Brazil).

Blood smears with anticoagulated samples were made and stained with Wright stain (Sigma‐Aldrich). Blood smears were evaluated using light microscopy (DM250, Leica Microsystems, Heerbrugg, Switzerland) to obtain leukocyte differential count, assess blood cell morphology, evaluate thrombocytes looking for clumped formations, and detect the presence of blood parasites or viral inclusions. A differential leukocyte count was performed by identifying 200 cells. Leukocytes were morphologically classified as basophils, heterophils, lymphocytes, azurophils, and monocytes [9, 18].

### 2.3 Biochemical Analysis

Serum separating tubes were centrifuged for 10 min at 2325 g to obtain serum (Centrifuge 80‐2B, Centrilab). Biochemical analytes, total protein, albumin, cholesterol, triglycerides, calcium, phosphorus, urea, creatinine, uric acid, aspartate aminotransferase (AST), alanine aminotransferase (ALT), alkaline phosphatase (ALP), gamma‐glutamyl transferase (GGT), and creatine kinase (CK) were measured using commercial kits (Laborlab) in an automated analyzer (CM‐250, Wiener Lab), previously calibrated (Laborcal, Laborlab) and checked with control serum containing known normal and pathological values (Laborcontrol 1 and 2, Laborlab). Globulin was calculated using a standard formula.

### 2.4 Statistical Analysis

Statistical procedures were performed according to ASVCP guidelines [19, 20]. All variables were assessed for normality using the D'Agostino‐Pearson test, which indicated a Gaussian distribution if *p* > 0.20. In the case of one or two outliers detected using Tukey's interquartile fences, these outliers were removed, even though the normality of the data distribution had been observed, before reassessing the normality of the data. If the removal of outliers resulted in a normal distribution, the analysis was considered complete. However, if the data still deviated from normality, the Box‐Cox transformation was applied to normalize the remaining data while preserving the original sample size. An outlier identified in the total leukocyte count was also excluded from the differential leukocyte analysis. When outliers were found in the relative leukocyte counts, the corresponding values were likewise removed from the absolute leukocyte counts. Descriptive statistics (mean, standard deviation, median, minimum, and maximum values) and RIs with 90% confidence intervals were calculated using MedCalc Statistical Software (MedCalc Software, Ostend, Belgium). The comparison of hematologic and biochemical variables was performed according to sex (female and male) and *Hepatozoon* spp. infection status (positive and negative). The Mann–Whitney test was used for variables with a non‐normal distribution, whereas ANOVA was applied to variables with a normal distribution.

## 3 Results

**FIGURE 1:**
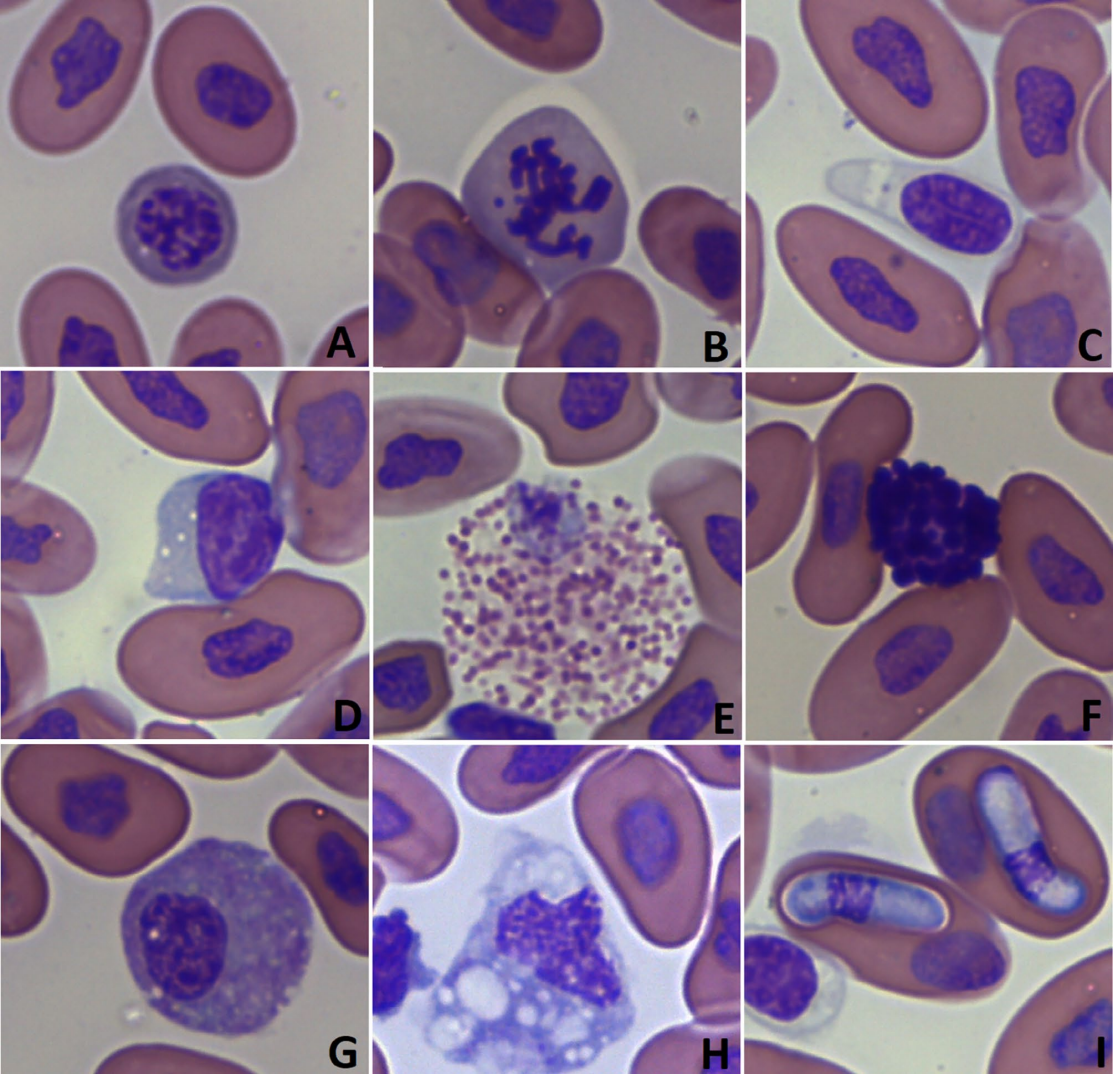
Photomicrographs showing the morphology of blood cells from *Bothrops jararaca* (A) polychromatophil, (B) mitotic figures in the erythroid cell, (C) thrombocyte, (D) lymphocyte, (E) heterophil, (F) basophil, (G) azurophil, (H) monocyte, and (I) intraerythrocytic gamonts of *Hepatozoon* spp. Wright Stain, 100× objective.

Erythrocytes were elliptical with eosinophilic cytoplasm and a basophilic, oval to pleomorphic nucleus. Fewer numbers of polychromatophils and rare mitotic figures in erythroid cell lines were observed (Figure 1A,B). Polychromatophils were rounder and more basophilic and had larger round or oval nuclei than mature erythrocytes, with higher nucleus‐to‐cytoplasmic ratios.

Thrombocytes were oval to round and with pale cytoplasm and a basophilic, oval to round nucleus, with condensed chromatin (Figure 1C). Rare small aggregates were observed in 12/32 blood smears.

Leukocytes were categorized into five cellular types: basophils, heterophils, lymphocytes, azurophils, and monocytes. Lymphocytes were the predominant leukocyte in *B. jararaca*. Lymphocytes were small blood cells with scant basophilic cytoplasm and a round nucleus (Figure 1D). Heterophils were large round cells with round to elongate, bright eosinophilic granules, and with round, eccentrically placed nuclei (Figure 1E). Basophils were round cells with round, intensely basophilic granules and also had a round, eccentrically placed nucleus (Figure 1F). Azurophils were the second most frequently observed leukocyte. These cells were round and contained numerous azurophilic granules and occasional vacuoles in the cytoplasm, with a round to oval nucleus (Figure 1G). Monocytes were rarely observed in most of the smears and were round, with a pale blue‐gray cytoplasm and with a round to oval nucleus (Figure 1H).

No viral inclusions were detected. Intraerythrocytic gamonts of *Hepatozoon* spp. were detected in blood smears of five female snakes (15%—5/32). Gamonts were elongated with a pale basophilic cytoplasm and an ovoid nucleus with dark purple chromatin, slightly displaced to one side of the parasite (Figure 1I).

**FIGURE 2:**
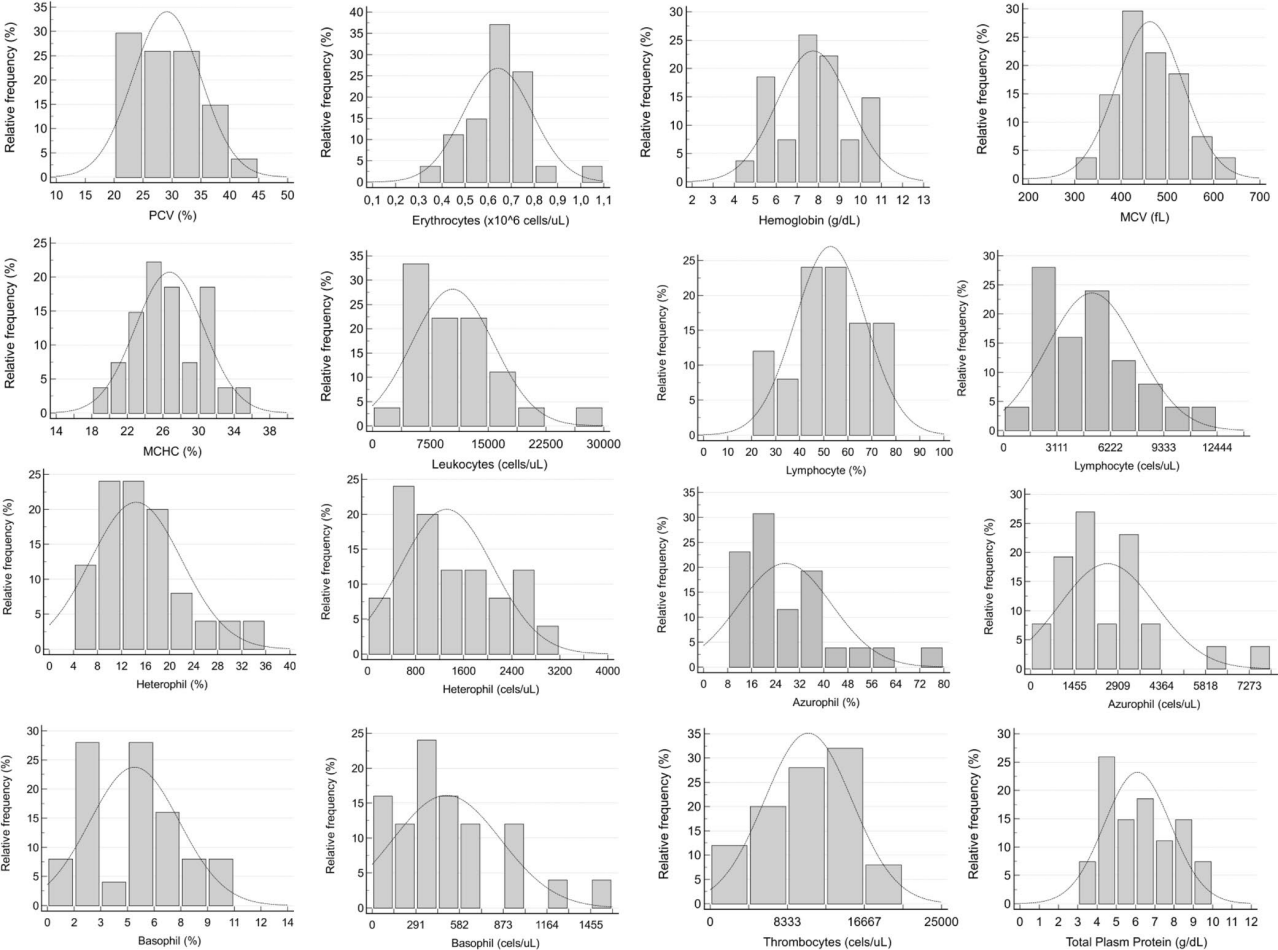
Histograms of the hematologic variables of Brazilian captive *Bothrops jararaca* representing relative frequency and normal distribution (dashed line). Gaussian distribution: erythrocytes, hemoglobin, MCV, MCHC, leukocytes, heterophils, lymphocytes, azurophil, basophil, thrombocytes. Non‐gaussian distribution: PCV, TTP.

**FIGURE 3:**
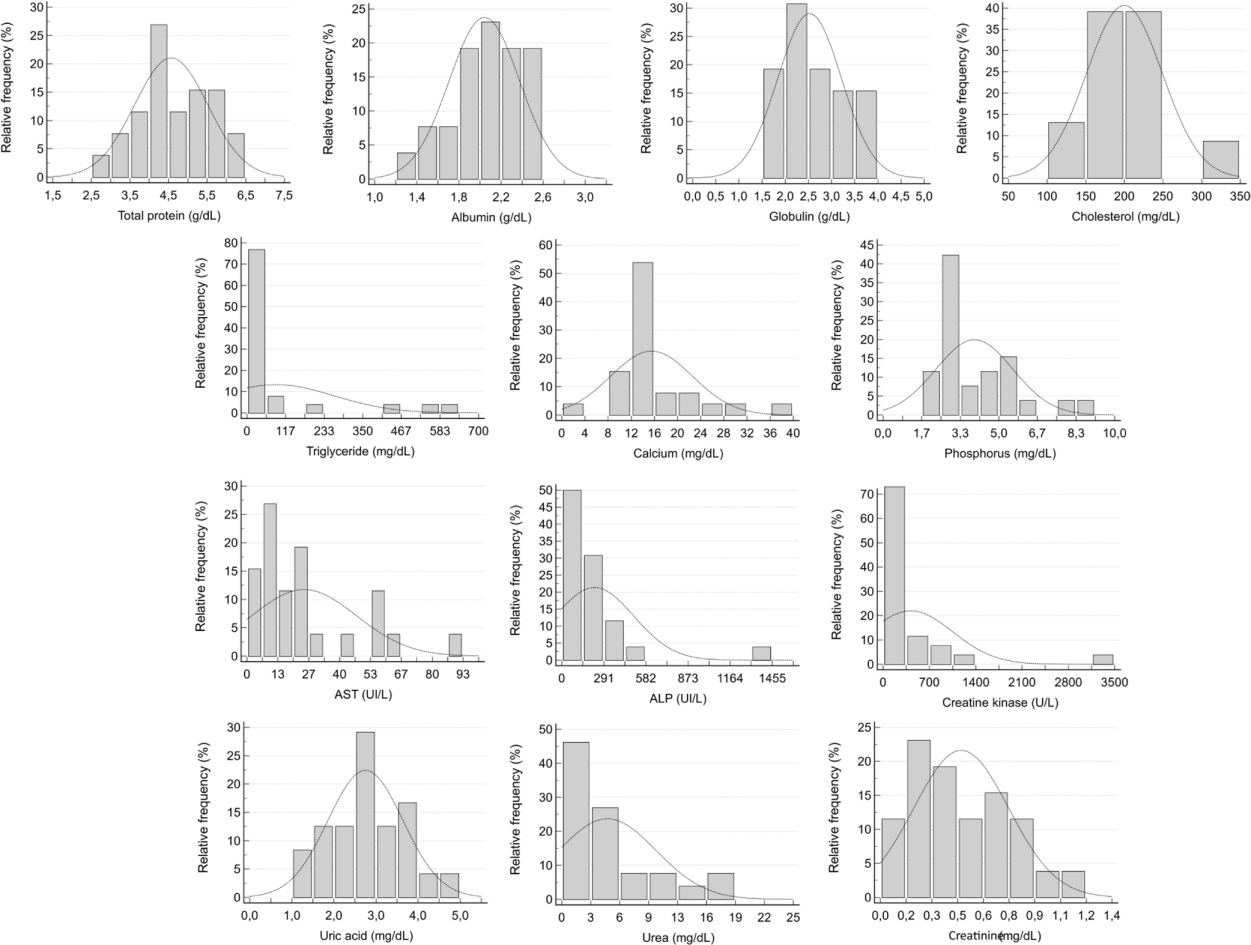
Histograms of the biochemical variables of Brazilian captive *Bothrops jararaca* representing relative frequency and normal distribution (dashed line). Gaussian distribution: phosphorus, uric acid, creatinine, AST, CK, ALP, triglyceride, cholesterol, total protein, albumin, Globulin. Non‐gaussian distribution: calcium, urea.

After excluding snakes infected with *Hepatozoon* spp. (*n* = 5), RIs for hematology and biochemistry were determined with 27 and 26 snakes, respectively. One snake in which *Hepatozoon* was detected did not have a sample for biochemical analysis. Outliers were identified and removed for the following variables: leukocytes (1), lymphocytes (2), heterophils (2), azurophils (1), basophils (2), thrombocytes (2), uric acid (2), and cholesterol (3). RIs for hematologic and biochemical variables are described in Tables 1 and 2. Histograms representing the relative frequency and normal distribution of these variables are shown in Figures 2 and 3.

**TABLE 1:** Descriptive statistics, 95% reference intervals plus confidence intervals (90%) of hematologic variables from captive *Bothrops jararaca* population.

Measurand	Conventional units	*N*	Mean	SD	Median	Min	Max	*p*	Dist	Method	LRL of RI	URL of RI	90% CI of LRL	90% CI of URL
PCV	%	27	29.1	5.9	28.0	20.0	40.0	0.190	NG	NP	20	40		
Erythrocytes	10^6^/μL	27	0.64	0.15	0.64	0.36	1.01	0.746	GS	P	0.35	0.93	0.27–0.43	0.85–1.02
Hemoglobin	g/dL	27	7.8	1.7	7.8	4.3	10.6	0.661	GS	P	4.4	11.1	3.4–5.3	10.2–12.1
MCV	fL	27	462.4	71.9	452.0	324.0	628.0	0.707	GS	P	321.4	603.3	281.4–361.3	563.4–643.3
MCHC	g/dL	27	26.7	3.9	26.5	18.7	34.8	0.926	GS	P	19.2	34.3	17.1–21.3	32.2–36.4
TPP	g/dL	27	6.1	1.72	6.00	3.60	9.40	0.138	NG	NP	3.2	9.4		
Leukocytes	10^3^/μL	26	10	4.17	8.74	3.36	18.80	0.392	GS	P	1.56	17.92	0–3.92	15.56–20.29
Heterophils	%	25	12.94		13.0	4.0	35.0	0.998	GS	PT	4.15	33.83	2.84–5.94	25.88–43.74
Heterophils	10^3^/μL	25	1.32	0.77	1.17	0.30	2.85	0.264	GS	P	1.02	10.31	0–0.25	2.38–3.27
Lymphocytes	%	25	52.8	14.77	54.0	25.0	79.0	0.782	GS	P	23.84	81.75	15.31–32.37	73.22–90.29
Lymphocytes	10^3^/μL	25	5.15	2.63	4.68	1.13	11.84	0.253	GS	P	1.02	10.31	0–1.15	8.79–11.83
Azurophils	%	26	23.17		23.0	10.0	74.0	0.816	GS	PT	9.27	72.05	7.33–11.87	50.46–100.0
Azurophils	10^3^/μL	26	2.24		2.05	0.37	7.38	0.831	GS	PT	0.48	6.60	0.26–0.80	4.98–8.56
Basophils	%	25	5.08	2.61	5.0	1.0	10.0	0.578	GS	P	0	10.2	0–1.47	8.69–11.71
Basophils	10^3^/μL	25	0.40		0.4	0.08	1.5	0.854	GS	PT	0.06	1.50	0.03–0.12	1.06–2.08
Thrombocytes	10^3^/μL	25	10.59	4.73	10.50	1.2	20.6	0.984	GS	P	1.3	19.86	0–4.05	17.13–22.60

Abbreviations: Dist, distribution; G, Gaussian; LRL, lower reference limit; MCHC, mean corpuscular hemoglobin concentration; MCV, mean cell volume; *N*, number of valid observations; NG, non‐Gaussian; NP, non‐parametric; P, parametric; PT, parametric transformed; RI, reference interval; TPP, total plasma protein; URL, upper reference limit.

**TABLE 2:** Descriptive statistics, 95% reference intervals plus confidence intervals (90%) of biochemical variables from captive *Bothrops jararaca* population.

Measurand	Conventional units	*N*	Mean	SD	Median	Min	Max	*p*	Dist	Method	LRL of RI	URL of RI	90% CI of LRL	90% CI of URL
Calcium	mg/dL	26	15.5	7.1	13.3	1.5	38.5	0.000	NG	NP	1.5	38.5		
Phosphorus	mg/dL	26	3.4		3.2	2.2	8.8	0.220	GS	PT	2.1	9.9	1.9–2.4	6.4–21.4
Uric acid	mg/dL	24	2.8	0.9	2.7	1.2	4.6	0.859	GS	P	1.0	4.5	0.5–1.5	4.0–5.0
Urea	mg/dL	26	4.9	5.3	4.0	0.0	18.0	0.030	NG	NP	4.0	18.0		
Creatinine	mg/dL	26	0.5	0.3	0.4	0.0	1.1	0.554	GS	P	0.0	1.1	0–0.1	0.9–1.2
AST	U/L	26	17.5		16.0	1.0	88.0	0.999	GS	PT	1.6	92.6	0.7–3.6	60.0–138.4
CK	U/L	26	203.5		201.0	59.0	3157.0	0.886	GS	PT	59.4	2896.2	45.6–79.9	960.2–23 700.8
ALP	U/L	26	145.4		145.4	63.7	1443.3	0.949	GS	PT	61.4	1021.1	51.2–75.5	444.1–6176.9
Triglyceride	mg/dL	26	17.5		18.0	1.0	640.0	0.978	GS	PT	0.8	885.9	0.4–1.8	255.4–3440.7
Cholesterol	mg/dL	23	200.1	49.1	198.0	111.0	316.0	0.359	GS	P	103.8	296.3	74.2–133.5	266.7–326.0
Total protein	g/dL	26	4.6	1.0	4.5	2.9	6.3	0.444	GS	P	2.7	6.4	2.2–3.3	5.9–7.0
Albumin	g/dL	26	2.0	0.3	2.0	1.3	2.5	0.619	GS	P	1.4	2.7	1.2–1.7	2.5–2.9
Globulin	g/dL	26	2.5	0.7	2.5	1.6	3.9	0.303	GS	P	1.2	3.9	0.8–1.6	3.5–4.3

Abbreviations: ALP, alkaline phosphatase; AST, aspartate aminotransferase; CK, creatine kinase; Dist, distribution; G, Gaussian; LRL, lower reference limit; *N*, number of valid observations; NG, non‐Gaussian; NP, non‐parametric; P, parametric; PT, parametric transformed; RI, reference interval; URL, upper reference limit.

Differences between females and males and infected and non‐infected with *Hepatozoon* spp. were not observed in hematologic and biochemical variables (Table 3).

**TABLE 3:** Comparison between male and female *Bothrops jararaca* hematologic and biochemical variables expressed as the mean (standard deviation).

Variable	Female (*n* = 25^a^, *n* = 23^b^)	Male (*n* = 7^a^, ^b^)	Infected (*n* = 5^a^, *n* = 4^b^)	Non‐infected (*n* = 27^a^, *n* = 26^b^)
Erythrocytes (10^6^/μL)^c^	0.66 (0.14)	0.6 (0.16)	0.58 (0.18)	0.64 (0.15)
Hemoglobin (g/dL)^c^	7.89 (1.46)	7.37 (2.4)	6.7 (1.4)	7.8 (1.7)
PCV (%)^c^	29.6 (5.9)	27.9 (6.0)	26.8 (6.1)	29.1 (5.9)
MCV (fL)^c^	456 (69.0)	480 (83)	477.7 (100.5)	462.4 (71.9)
MCHC (%)^c^	27.0 (3.5)	26.1 (4.9)	25.0 (3.3)	26.7 (3.9)
Leukocytes (10^3^/μL)^c^, ^d^	10.3 (5.8)	10.62 (4.1)	13.3 (6.1)	10.4 (5.3)
Heterophils (10^3^/μL)^d^	1.5 (1.0)	2.2 (2.8)	1.5 (0.7)	1.7 (1.7)
Lymphocytes (10^3^/μL)^c^, ^d^	5.7 (3.8)	4.7 (2.9)	7.4 (3.6)	5.4 (3.5)
Azurophils (10^3^/μL)^d^	2.5 (1.4)	3.1 (2.5)	3.6 (2.0)	2.7 (1.7)
Basophil (10^3^/μL)^d^	0.64 (0.68)	0.47 (0.23)	0.8 (0.65)	0.6 (0.59)
Monocytes (10^3^/μL)^d^	0.03 (0.09)	0.04 (0.11)	0	0.03 (0.03)
Thrombocytes (10^3^/μL)^c^, ^d^	11.9 (5.7)	12.2 (9.9)	13.9 (8.4)	12.0 (6.8)
TPP (g/dL)^c^	6.3 (1.7)	5.4 (1.7)	5.3 (1.8)	6.1 (1.7)
Total protein (g/dL)^c^	4.6 (0.8)	4.4 (1.3)	4.3 (0.3)	4.6 (1.0)
Albumin (g/L)^c^	2.1 (0.3)	2.0 (0.5)	1.9 (0.1)	2.0 (0.3)
Globulin (g/dL)^c^	2.6 (0.7)	2.4 (0.8)	2.4 (0.2)	2.5 (0.7)
Cholesterol (g/dL)^d^	220 (100)	188 (35)	173.5 (33.1)	214.0 (87.1)
Triglycerides (g/dL)^d^	107 (199)	34 (65)	91.3 (79.6)	87.5 (200.5)
Calcium (mg/dL)^d^	15.9 (7.6)	14.5 (5.7)	13.9 (0.7)	15.5 (7.1)
Phosphorus (mg/dL)^d^	4.0 (1.7)	3.7 (1.7)	3.8 (0.5)	5.9 (3.5)
AST (U/L)^c^, ^d^	27.9 (23.5)	15.9 (19.0)	15.0 (12.1)	24.7 (22.7)
ALT (UI/dL)^d^	2.2 (4.3)	0.7 (1.9)	0	1.8 (3.8)
ALP (U/L)^c^	228 (311)	221 (132)	225.2 (28.3)	226.2 (272.0)
GGT (U/L)^d^	3.4 (5.1)	3.1 (5.2)	3.9 (4.5)	3.3 (5.0)
CK (U/L)^d^	492 (726)	213 (190)	234.3 (168.2)	417.1 (635.4)
Uric acid (mg/dL)^d^	3.2 (2.3)	3.6 (2.3)	2.4 (0.8)	3.3 (2.2)
Urea (mg/dL)^d^	4.3 (5.1)	6.7 (5.6)	2.5 (2.4)	4.9 (5.3)
Creatinine (mg/dL)^c^	0.48 (0.28)	0.50 (0.32)	0.46 (0.19)	0.49 (0.29)

Abbreviations: ALT, alanine aminotransferase; AST, aspartate aminotransferase; CK, creatine kinase; GGT, gamma‐glutamyl transferase; MCHC, mean corpuscular hemoglobin concentration; MCV, mean cell volume; PCV, packed cell volume; TPP, total plasma protein.

^a^ Number of animals for hematological variables.

^b^ Number of animals for biochemical variables. *p‐*values: All comparisons yielded *p* > 0.05.

^c^ Parametric method (ANOVA).

^d^ Non‐parametric method (Mann–Whitney test).

## 4 Discussion

Establishment of RIs for CBC and clinical chemistry analytes is an important tool for conservation and monitoring the health of captive snakes. This is the first study that provides population‐based RI for *B. jararaca* .

Considering the PCV and erythrocyte counts, *those of B. jararaca* were similar to those of other species of *Bothrops* and *Crotalus* genus [5, 6, 7, 8, 21]. Hemoglobin concentration was similar to *B. asper* , *B. pubescens* , and *Crotalus sinus* [8, 21], but lower than values found in some *Bothrops* snakes [5]. Rare polychromatophils were observed in *B. jararaca* . Fewer numbers of immature erythrocytes have been reported in healthy *B. pubescens* [8].

Regarding the leukocyte counts, the mean values *of B. jararaca* were similar to *Bothrops alternatus, Bothrops jararacussu, Bothrops moojeni, Bothrops neuwiedii*, and *Crotalus durissus* [5, 22], but were higher than in *Bothrops leucurus* and *Bothrops asper* [6, 21]. Lymphocytes were the most prevalent leukocyte in *B. jararaca* snakes in our study, as previously reported in other *Bothrops* spp. snakes [5, 6, 7, 8, 21].

Azurophils were the second most frequent leukocyte in *B. jararaca* . These cell types are usually observed in snakes and occasionally detected in lizards, chelonians, and crocodilians and are also morphologically and functionally resembling both granulocytes and monocytes [9, 18]. Snake azurophils have a cytochemical composition like that of mammalian neutrophils, whereas lizard azurophils are similar to mammalian monocytes. Consequently, the authors recommend counting azurophils separately in snakes, while grouping them with monocytes in other reptile species [9, 18].

Few monocytes were observed in the present study. In other species, such as *B. asper, B*. *alternatus* , *B. jararacussu*, and *B. moojeni*, higher monocyte concentrations were detected [5, 21]. These cells were not detected in *Bothrops pubescens, B. jararacussu*, and *B. leucurus* [7, 8].

Eosinophils were not observed in *B. jararaca*, as previously described in snakes of the *Bothrops* genus, such as *B. pubescens* , *B. jararacussu* , and *B. leucurus* [6, 7, 8], but it differs from *B. asper* [21].

Considering the biochemical variables, uric acid (UC) concentration in *B. jararaca* was similar to those of other species of the *Bothrops* genus [7, 8, 21]. Creatinine and urea concentrations in *B. jararaca* were close to those found in *C. durissus* [23]. Mean values of calcium and phosphorus were resplendent in *B. asper* and *C. durissus* [21, 23].

In our study, the protein concentration was determined using the refractometric and biuret methods, and higher TTP values were detected. A correlation between protein estimation using refractometry and the biuret method is expected [24]; however, significant discrepancies between the biuret and refractometric results may arise from the interference of non‐protein solids such as cholesterol, urea, lipoproteins, and glucose [24]. Considering the protein fractions, the mean albumin and globulin concentrations found in *B. jararaca* were like those of other species of the *Bothrops* genus [7, 8, 21].

AST activity is higher than ALT activity in reptiles, as observed in *B. jararaca* and previously in *C. durissus* [ 23]. However, AST activity in *B. jararaca* was lower than the results found in *B. asper, B. pubescens*, *and C. durissus* [8, 21, 23]. Our results for serum CK activity were similar to those found in *B. asper* and *B. pubenscens* [18, 21]. Cholesterol and triglyceride values were alike in *B. asper* [21].

In our study, the evaluation of hemogregarines was limited to microscopic analyses, which identified *Hepatozoon* spp. in five female snakes. Molecular techniques, in addition to greater sensitivity, could be used for species‐level identification [12]. No significant differences in any hematologic or biochemical analytes were detected in wild‐caught pythons (*Morelia imbricata*) from Australia according to hemogregarine parasite status [14]. In neotropical wild‐caught rattlesnakes ( *C. durissus* ) from Brazil, animals infected with *Hepatozoon* spp. exhibit a higher polychromatophilic percentage than non‐infected snakes [22]. Regarding morphology features, in a morphometric evaluation of erythrocytes infected and non‐infected with *Hepatozoon* gamonts from *Bothrops* snakes, no differences were observed in the size of infected erythrocytes [25]. However, gametocytes were often found displacing the nucleus to the periphery of *B. jararaca* erythrocytes, as previously observed in infected snakes [25].

The ASVCP guidelines recommend a minimum of 120 individuals for accurate RI estimation, but this sample size is often difficult to achieve in veterinary medicine, especially for wild animals. Although small sample sizes limit precision and accuracy, they are still important for the clinical evaluation of *B. jararaca* specimens in captive conditions. Additionally, the small sample size reduces the power of comparisons between hematologic and biochemical variables related to sex and *Hepatozoon* status.

Our study provides the first RI for hematologic and biochemical variables for *B. jararaca*. RIs determined from smaller sample sizes are commonplace and often necessary in veterinary medicine, especially in wild animals, as in our study. The limitations of our findings are related to a small sample size and the absence of comparison between different environmental conditions. Using RIs, species specificity is important for reliable interpretation since there are differences even in related species. It is an important tool for monitoring captive *B. jararaca* in similar conditions of environment. No difference was observed between male and female variables; however, it is important to consider the small sample size, especially of males, in our study.

## Conflicts of Interest

The authors declare no conflicts of interest.

## Supporting information


**Table S1:** vcp70032‐sup‐0001‐TableS1.docx.
