# Supplementary material for: Hematology, Clinical Biochemistry, and Blood Cell Morphology Features of Captive Bothrops jararaca From Southeast Brazil
Source: Vet Clin Pathol. 2025 Aug 25;54(3):221–9. doi: 10.1111/vcp.70032 (PMC12444004; doi:10.1111/vcp.70032)
Supplement: Supplementary file 1 — Table S1: vcp70032‐sup‐0001‐TableS1.docx. [file VCP-54-221-s001.docx]

**Supplementary Table.** Methods used for biochemistry analysis

| Analyte | Method |
| --- | --- |
| Total protein (g/dL) | Colorimetric (Biuret) |
| Albumin (g/dL) | Colorimetric (Bromocresol green) |
| Globulin (g/dL) | Calculated (Protein − Albumin) |
| Cholesterol (mg/dL) | Enzymatic (CHOD/POD) |
| Triglycerides (g/dL) | Enzymatic (GPO/PAP) |
| Calcium (g/dL) | Colorimetric (Arzenazo III) |
| Phosphorus (U/L) | Colorimetric (Phospomolybdate) |
| AST (U/L) | UV Kinetic (IFCC) |
| ALT (U/L) | UV Kinetic (IFCC) |
| ALP (U/L) | Kinetic (DGKC) |
| CK (U/L) | UV Kinetic |
| GGT (U/L) | Colorimetric (Szasz) |
| Uric acid (mg/dL) | Enzymatic (UOD/POD) |
| Urea (mg/dL) | Enzymatic (Urease/GLDH) |
| Creatinine (mg/dL) | Colorimetric (Alkaline Picrate - Jaffé) |

Abbreviations: ALP, alkaline phosphatase; ALT, alanine aminotransferase; AST, aspartate aminotransferase; CK, creatine kinase; GGT, gamma-glutamyl transferase.
